# Dosimetric evaluation of systematic source position uncertainty along the source path and robust planning during MRI‐only cervical HDR brachytherapy

**DOI:** 10.1002/acm2.70810

**Published:** 2026-09-23

**Authors:** Amritpal Singh, Raffi Karshafian, Ananth Ravi, Moti Raj Paudel

**Affiliations:** ^1^ Department of Physics Toronto Metropolitan University Toronto Ontario Canada; ^2^ Institute for Biomedical Engineering, Science and Technology (IBEST) Toronto Ontario Canada; ^3^ Department of Medical Physics Sunnybrook Health Science Centre Toronto Ontario Canada; ^4^ Department of Radiation Oncology University of Toronto Toronto Ontario Canada

**Keywords:** cervical cancer, high‐dose‐rate, interstitial brachytherapy, intracavitary, MRI

## Abstract

**Background:**

The knowledge of the dosimetric impact of systematic source position uncertainty and the use of a methodology for robust treatment planning against such uncertainty can help improve the quality of treatment plans, during MR‐only cervical brachytherapy treatment.

**Purpose:**

To assess the dosimetric impact of systematic source position uncertainty along the source path and evaluate strategies to minimize it during MRI‐only cervical high‐dose‐rate (HDR) brachytherapy treatment planning.

**Methods:**

Treatment plans from 22 cervical cancer patients (FIGO stages IB2–IVA; 11 intracavitary brachytherapy (ICBT), 11 interstitial brachytherapy (ISBT)) were retrospectively analyzed. Positional uncertainty was modeled by systematically shifting the source position (± 1 to ± 5 mm with 0 mm as the reference position) within applicators along the source path in the original plans. Dosimetric parameters, HRCTV_D90 and D_2cc_ for organs at risk (OARs), were computed for each source position. Statistical significance was assessed via paired t‐test and Wilcoxon signed‐rank test. Targeted plans exceeding dosimetric limits for OARs were re‐optimized by modifying dwell times or gradients within 1 cm of the targeted OAR to improve the variability and overall OAR dosimetry.

**Results:**

Significant dosimetric changes were observed for bladder, sigmoid (all shift positions; *p *≤ 0.001) and rectum (1 to 5 mm; *p* ≤ 0.04) in ICBT, and for all OARs (all shift positions; *p* ≤ 0.003) in ISBT. HRCTV_D90 was significantly affected in ICBT from ‐2 to ‐5 mm (*p* ≤ 0.042) and at all shifts (*p* ≤ 0.033) except at ‐1 and ‐2 mm in ISBT. Within ± 5 mm shift range, the mean (maximum) drop in HRCTV_D90 and increase in OARs (bladder, bowel, rectum and sigmoid) D_2cc_ were up to −2.0 ± 1.9(−5.4)% and (6.0 ± 3.4(13.6)%, 0.5 ± 2.5(5.5)%, 2.2 ± 2.7(7.4)% and 1.8 ± 1.2(4.2)%) for ICBT, and ‐6.2 ± 1.4(‐11.9)% and (5.2 ± 4.6(17.2)%, 1.3 ± 0.9(3.0)%, 4.6 ± 2.5(7.6)% and 6.6 ± 3.6(13.8)%) for ISBT, respectively. Re‐optimized plans showed improved robustness of dosimetry against source positional uncertainty for the targeted OAR with the same or better dosimetry for other OARs and HRCTV. The strategies to obtain this robustness were patient and applicator‐type dependent.

**Conclusion:**

Systematic source position uncertainty significantly influences dosimetry in MRI‐only cervical HDR brachytherapy. Adjusting dwell times and gradients near the targeted OAR could minimize this effect.

## 1 INTRODUCTION

The combination of external beam radiation therapy (EBRT), chemotherapy, and brachytherapy (BT) is the state‐of‐the‐art treatment for locally advanced cervical cancer.^1^, ^2^ Globally, 52%‐81% of cervical cancer patients receive BT along with EBRT.^3^, ^4^, ^5^ Modern three‐dimensional (3D) imaging‐based BT employs computed tomography (CT), magnetic resonance imaging (MRI), or a hybrid CT/MRI treatment approach.^6^, ^7^, ^8^ More recent advancements have led to the development of MRI‐only high‐dose‐rate (HDR) BT, enabling target/organs at risk (OARs) delineation and applicator reconstruction in magnetic resonance (MR) images.^9^, ^10^, ^11^

During MRI‐only cervical BT, dose delivery accuracy depends on the consistency of source position within the applicator and applicator position within the patient relative to the target and OARs between treatment planning and delivery. Researchers have estimated the applicator shift^12^, ^13^ and dosimetric effects of the applicator displacements.^14^, ^15^, ^16^

Source position uncertainty within the applicator affects HDR BT dosimetry, which depends on the type and design of the applicator. Straight applicators like tandems can have source position uncertainty of up to 2.3 mm.^17^, ^18^ Inside curved applicators like ring or ovoid, the source is pushed toward the outer wall within the applicator lumen,^19^ leading to a non‐circular path. Non‐circular motion of the source may result in a deviation of source position compared to source motion in a straight applicator.^20^ Source position uncertainty has been seen to range from 2.5 to 4.5 mm in ring applicators.^21^, ^22^, ^23^ Venezia (Elekta AB, Stockholm, Sweden) is a relatively new tandem and ovoid applicator used in cervical HDR BT. Source position uncertainty can range from 0.2 to 2.1 mm in the Venezia applicator.^24^, ^25^ Additionally, over the lifecycle of a source drive cable, its length can change up to 1 mm.^23^

On standard T2‐weighted MR images, interstitial brachytherapy catheters appear as low‐signal‐intensity regions making it challenging to distinguish them from surrounding soft tissues or voids without the use of markers. However, for workflows using T1‐weighted MR images along with liquid‐filled MR‐line markers, air pockets or bubbles can ascend to the tip of the line marker/catheter, resulting in reconstruction uncertainties at the tip of the catheter.^9^ The reconstruction uncertainty of an applicator tip in MR images during MR‐only brachytherapy treatment planning has been observed to be up to 3.3 mm,^9^, ^26^, ^27^ which results in an equivalent uncertainty in the source position within the applicator. The dose gradient in BT often varies from 5% to 12% per mm at distances of 1 to 3 cm from the source.^11^ High dose gradients in HDR BT necessitate an investigation of the dosimetric impact of source position uncertainty for each applicator.

Previous studies mentioned above^12^, ^13^, ^14^, ^15^, ^16^ have not focused on the robustness of cervical HDR brachytherapy treatment plans; rather, have mainly studied the dosimetric change of applicator shift as an uncertainty. To our knowledge, no study in the literature has presented ways to increase the dosimetric robustness of treatment plans in the presence of source position uncertainty within applicators for MR‐only cervical HDR brachytherapy. Our study assesses the dosimetric effect of systematic source position uncertainty, introduced as a shift along the source path in a small range of ± 5 mm, and evaluates strategies to minimize it for MRI‐only cervical HDR brachytherapy.

## 2 METHODS

### 2.1 Patient selection

Our study included 22 randomly and retrospectively selected cervical cancer patients treated with MRI‐only HDR brachytherapy. These patients, with FIGO stages from IB2 to IVA, were treated between January 2020 and September 2022 at our institution. The study has been approved by applicable research ethics boards. Selected patients were subdivided into two groups based on the treatment technique used. In group 1 (11 patients), patients received intracavitary brachytherapy (ICBT), and in group 2 (11 patients), patients received interstitial brachytherapy (ISBT). ICBT treatments were delivered by using the Venezia applicator, and ISBT treatments were delivered by using a transperineal interstitial template with tandem and plastic ProGuide (Elekta AB, Stockholm, Sweden) needles.

### 2.2 Data collection

All patients had been treated with combined EBRT (4500cGy in 25 daily fractions) followed by three intraoperative HDR BT boost treatments. For HDR BT treatment, each patient was scanned in a 1.5T MR scanner (Ingenia 1.5T, Philips Healthcare, Netherlands), and multiparametric MR images were acquired for treatment planning using various image sequences, including T2‐weighted 3D (T2W‐3D), T1‐weighted 3D (T1W‐3D), and T2‐weighted 2D (T2W‐2D). These co‐registered images were used to contour the target and OARs in MIM (MIM Inc. Cleveland, USA), with the T1W‐3D image used as the primary image for catheter reconstruction and dose calculation. Plan optimization and dosimetric calculations were completed in the Oncentra Brachy (Elekta AB, Stockholm, Sweden) treatment planning system (TPS). The plan quality was ensured following the EMBRACE II recommendations for target coverage, OARs sparing, and treatment plan robustness.^28^ To quantify the dosimetric impact of systematic source position uncertainty, dose volume histogram (DVH) parameters: D90 for high‐risk clinical target volume (HRCTV) and minimum dose in the most irradiated 2cc tissue volume (D_2cc_) for bladder, bowel, rectum, and sigmoid, were used following GEC‐ESTRO guidelines.^29^ These parameters are summarized in Table 1.^30^

**TABLE 1:** Dose metric for target (HRCTV) and organs at risk according to EMBRACE II.

Dose metric	Definition	Planning aims^*^EQD_2_(Gy)	Planning limits EQD_2_(Gy)
HRCTV_D90	Minimum dose delivered to 90% of HRCTV volume	>90, < 95	> 85
Bladder D_2cc_	Minimum dose in the most irradiated 2cc tissue volume of the bladder	< 80	< 90
Bowel D_2cc_	Minimum dose in the most irradiated 2cc tissue volume of the bowel	<70	<75
Rectum D_2cc_	Minimum dose in the most irradiated 2cc tissue volume of the rectum	<65	<75
Sigmoid D_2cc_	Minimum dose in the most irradiated 2cc tissue volume of the sigmoid	<70	<75

* EQD_2_: Biologically equivalent dose in a 2 Gy/fraction schedule in the linear quadratic model.

**FIGURE 1:**
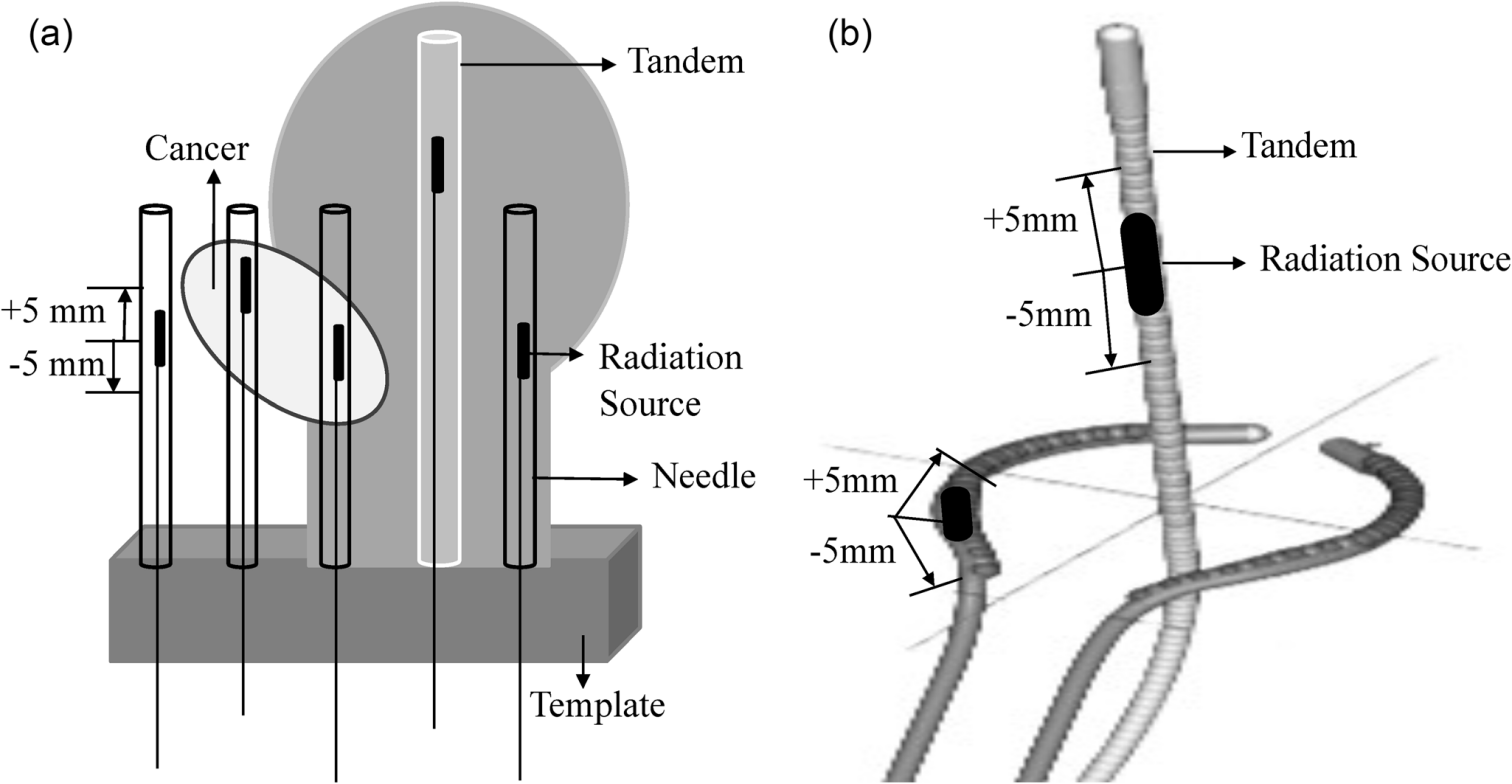
Schematic diagram of (a) interstitial brachytherapy using a template, needles and tandem (b) intracavitary brachytherapy using Venezia tandem and ovoid applicators showing positive and negative source shifts along the source movement path.

For ICBT patients, the applicator was delineated, and the source path within the applicator was reconstructed using applicator library models present in the Oncentra Brachy TPS from the originally delivered treatment plan. The source path, dwell positions, and dwell times were copied from the existing delivered treatment plan in the Oncentra Brachy. Systematic source position shifts within applicators were introduced by maintaining the dwell times and changing the dwell position in steps of 1 mm from 0 to ± 5 mm (Figure 1b) using the ‘Offset’ feature within Oncentra Brachy TPS for the source channels of the tandem and ovoids. The 0 mm shift corresponded to the original treatment plan and was taken as the reference source position. For tandem and ovoids, the sign conventions were defined such that a positive (+) uncertainty corresponds to a distal shift (towards the tip end of the applicator) and a negative (−) uncertainty corresponds to a proximal shift in source position (towards the connection end of the applicator). Finally, DVH parameters (physical dose for HRCTV and OARs) were obtained for each systematically shifted source position from the Oncentra Brachy. These parameters were converted to the EQD_2_ for each source position (separately for each structure of interest) for the BT fraction (EQD_2BT_) schedule using the following formula.^31^

(1) $$\mathrm{EQ}D_{2\mathrm{BT}}={\mathrm{Nd}}^{*}\frac{\left(1\;+\;\frac{d}{\alpha /\beta }\right)}{\left(1\;+\;\frac{2}{\alpha /\beta }\right)}$$

where N, d, α/β are the number of fractions, the dose per fraction for each structure of interest obtained from the Oncentra Brachy, and the parameters in the linear‐quadratic cell survival model. The value of α/β is taken to be 3 and 10 for OAR and HRCTV, respectively. There was no overlap of target volume with critical structures for any of the patients studied, and therefore EQD_2_ for each volume was calculated independently without any priority rule.

For the purpose of the study, it was assumed that the same dose was delivered in all BT fractions. Additionally, the EQD_2_ from EBRT (EQD_2EBRT_) was added to EQD_2BT_ to obtain the total biologically equivalent dose (EQD_2_).

For ISBT patients, the source path was digitized manually for tandem and needles in the originally treated plan. The original plan in the Oncentra Brachy was copied, and the uncertainty in the source position inside the source channel of the tandem and needles was introduced in the same way as mentioned above. The same dosimetric parameters were read and converted to EQD_2_, similar to ICBT patients. Both for ICBT and ISBT, the percentage change in each doseimtric parameter (DP) for each shifted source position (from −5 to +5 mm) was calculated with respect to the reference source position by using the following equation:

(2) $$\begin{array}{cc} & \mathrm{Percentage}\;\mathrm{change}\;\mathrm{in}\;\mathrm{the}\;\mathrm{DP}\;=\;100^{*}\\ & \frac{\left(DP_{\mathrm{Shifted}\;\mathrm{source}\;\mathrm{position}}-DP_{\mathrm{Reference}\;\mathrm{source}\;\mathrm{position}}\right)}{\left(DP_{\mathrm{Reference}\;\mathrm{source}\;\mathrm{position}}\right)} \end{array}$$

### 2.3 Statistical analysis

Statistical analysis was performed using SPSS (IBM SPSS Statistics, Version 29.0.2.0 (20)) statistical analysis software. The variation of HRCTV_D90 and D_2cc_ for the bladder, bowel, rectum, and sigmoid, as well as their (HRCTV_D90 and D_2cc_) gradient with respect to the systematic source position shift, were evaluated. For graphical representation of dosimetric variations Box‐and‐Whisker plots were generated. The statistical significance of DVH parameter variation for the HRCTV and OARs with the source position uncertainty was tested using the paired t‐test and the Wilcoxon signed‐rank test for patients treated using ICBT and ISBT, respectively. The plans with 0 mm shift in the source position were taken as the reference plans. Normality of the dosimetric data was assessed using the Shapiro‐Wilk test (IBM SPSS Statistics, Version 29.0.2.0 (20)). Due to the normality of the data, the paired t‐test was used for ICBT. However, due to observed non‐normality, the non‐parametric Wilcoxon signed‐rank test was employed for all paired comparisons in ISBT. A *p* value < 0.05 indicated a statistically significant difference.

### 2.4 Re‐optimization of treatment plans

Re‐optimization was done on treatment plans for patients with the OAR D_2cc_ exceeding the planning aim or limit by the most in the presence of uncertainty. In the re‐optimized plan, it was aimed to reduce the OAR D_2cc_ while preserving target coverage. The impact of re‐optimization was quantified as the percentage change in HRCTV_D90 and OARs D_2cc_ relative to the original plan. The percentage change in a dosimetric parameter (DP) for a given source position uncertainty was calculated using the following equation:

(3) PercentagechangeintheDP=100∗DPRe−optimizedplan−DPOriginalplanDPOriginalplan

The re‐optimization also followed the EMBRACE II recommendations.

(a) Re‐optimization strategy for ICBT treatment plans

In ICBT reference plans, the planning process starts with a standard library plan in which a uniform loading of tandem and ovoids with a fixed dwell time ratio between them exists. The plan is further optimized by first creating modulation in tandem dwell times at the level of D_2cc_ for the OAR exceeding dosimetric goals. These dwells most often correspond to the midway locations of the tandem dwells. The ovoid dwells are often uniform. In our targeted OAR dose reduction strategy, further modulation was applied to the ovoid applicator. This often resulted in lowering dwell times at the proximal dwell positions with respect to the concerned OAR within 1 cm of them. Due to the anatomical location of the bowel loops, bowel sparing required a more sophisticated, multi‐applicator strategy. Besides reducing dwell times in ovoids within 1 cm to bowel dwell times in the tandem at the level of bowel D_2cc_ were reduced. To maintain target coverage for all re‐optimized plans, dwell times were redistributed to the dwell positions within the tandem and ovoids away from the OAR, where feasible.

(b) Re‐optimization strategy for ISBT treatment plans

The re‐optimization criterion involved minimizing dwell time and minimizing or removing modulation from dwell positions within 1 cm of the targeted OAR. For the OAR in which the source path within 1 cm is running along the OAR contour at the level of D_2cc_, dwell positions remain at a constant distance from the OAR in the presence of source position uncertainty. In this scenario, reducing or even removing modulation in the applicators within 1 cm with fixed lower dwell times was effective. For the source path approaching the OAR contours, dwell positions could be closer to the OAR in the presence of source position uncertainty. In this situation, creating more modulation with a reduction in dwell times closer to the OAR was effective. The reduction in target dose in either scenario was compensated by redistributing dwell weights to other dwell positions of tandem and/or neighboring needles that were not critical for OAR's D_2cc_.

### 2.5 Use of generative AI tools in the manuscript preparation

During the preparation of this research article the author(s) used Google Gemini to improve readability. After using this tool/service, the author(s) reviewed and edited the content as needed and take(s) full responsibility for the content of the published article.

## 3 RESULTS

**FIGURE 2:**
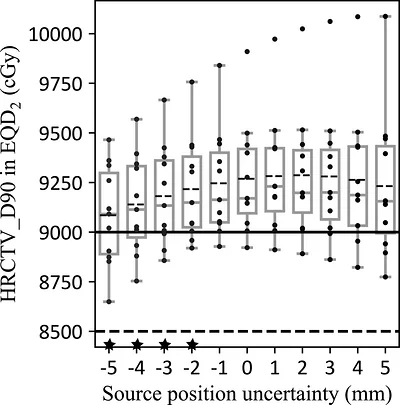
Uncertainty in HRCTV_D90 (EQD_2_) as a function of systematic source position uncertainty within ovoid and tandem applicators for intracavitary brachytherapy. The box of each plot with a 50% patient population represents the interquartile range. Data points outside of whiskers are considered outliers. The solid and dotted lines within each box indicate the median and mean values of the dosimetric parameter, respectively. The dashed and solid lines in the figures represent the limit for dose prescription and planning aim, respectively. Source positions corresponding to statistically significant dosimetric variations are marked with a star. Details regarding plot construction and symbols are provided here and apply to subsequent Figures 3, 4, 5.

**FIGURE 3:**
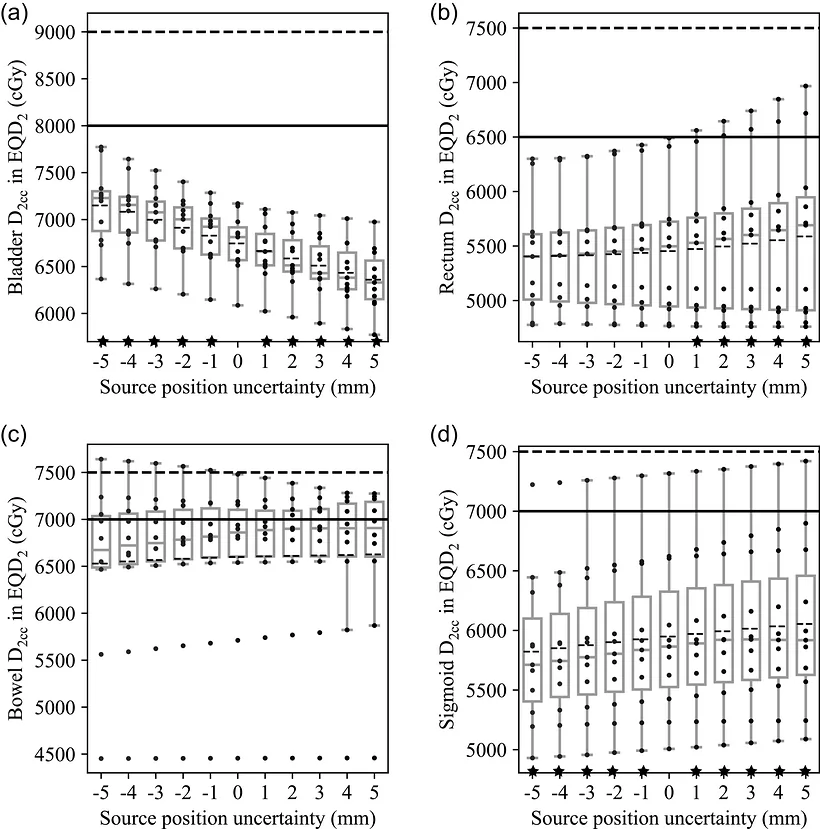
Uncertainty in D_2cc_ (EQD_2_) in intracavitary brachytherapy as a function of systematic source position uncertainty within ovoid and tandem applicators for: (a) bladder, (b) rectum, (c) bowel, and (d) sigmoid.

**FIGURE 4:**
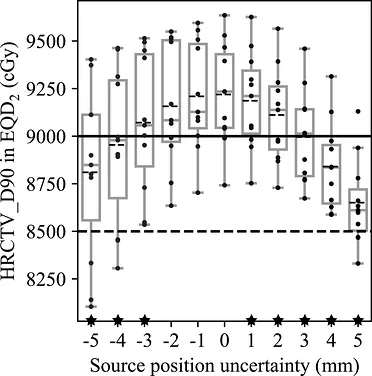
Uncertainty in HRCTV_D90 (EQD_2_) as a function of systematic source position uncertainty within interstitial needles and tandem applicators for interstitial brachytherapy.

**FIGURE 5:**
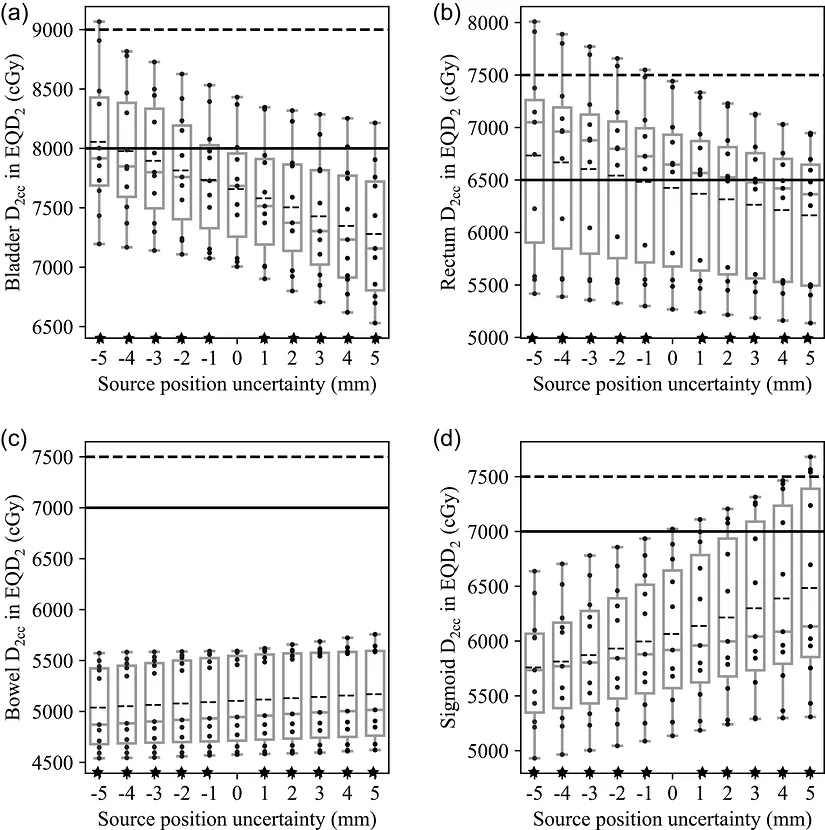
Uncertainty in D_2cc_ (EQD_2_) in interstitial brachytherapy as a function of systematic source position uncertainty within interstitial needles and tandem applicators for: (a) bladder, (b) rectum, (c) bowel, and (d) sigmoid.

The dosimetric variations with systematic source position uncertainties are shown in Figures 2 and 3 for the ICBT and Figures 4 and 5 for the ISBT treatments. The average HRCTV_D90 varied nonlinearly for ICBT with the systematic source position uncertainty (Figure 2), and it increased with an increase in source position uncertainty from 0 to +2 mm. An average increase of 0.4 ± 0.7% was observed at +2 mm shift in source position. The variation of HRCTV_D90 was statistically significant (*p* ≤ 0.042) at the systematic source position shift of −2 to −5 mm. An average dosimetric uncertainty of −2.0 ± 1.9% and −0.4 ± 2.1% was observed at −5 and +5 mm, respectively, with the largest drop of 5.4% for an individual patient at −5 mm. Moreover, four, one, and three patients did not meet the HRCTV_D90 planning aim for systematic source position uncertainties of −5, 0, and +5 mm, respectively.

The variation of D_2cc_ with systematic source position uncertainty in ICBT for OARs is shown in Figure 3. Bladder D_2cc_ (Figure 3a) decreased with an increase in positive source position shifts, whereas it increased for other OARs [rectum (Figure 3b), bowel (Figure 3c), and sigmoid (Figure 3d)] with the exception of one patient for bowel. The D_2cc_ variation was statistically significant (*p* ≤ 0.001) at all source position uncertainties for bladder and sigmoid, and at only positive uncertainties for rectum (*p* ≤ 0.04). The D_2cc_ variation for bowel was not statistically significant (*p* ≥ 0.197). The mean D_2cc_ gradient was ‐85cGy/mm, 10cGy/mm, 18cGy/mm, and 23cGy/mm for bladder, bowel, rectum, and sigmoid, respectively. The D_2cc_ changed by 6.0 ± 3.4%, −1.1 ± 2.3%, −0.7 ± 1.7%, and −2.1 ± 1.1% at −5 mm and −5.7 ± 2.1%, 0.4 ± 2.5%, 2.2 ± 2.7%, and 1.8 ± 1.2% at +5 mm uncertainty for bladder, bowel, rectum, and sigmoid, respectively. The largest increase in D_2cc_ for bladder was 13.6% (at −5 mm), whereas it was 5.5%, 7.4%, and 4.2% for bowel, rectum, and sigmoid, respectively, at +5 mm. Moreover, the D_2cc_ increased above the planning aim at +2 mm source position uncertainty for rectum (two patients) and above the planning limit at −1 mm for bowel (one patient).

The average HRCTV_D90 varied nonlinearly for ISBT treatments with the systematic source position uncertainty (Figure 4). The variation of HRCTV_D90 was statistically significant (*p* ≤ 0.033) at all source positions except at −1 and ‐2 mm. An average dosimetric uncertainty of −4.5 ± 3.7% and −6.2 ± 1.4% was observed at −5 and +5 mm, respectively, with the largest drop of 11.9% for an individual patient at −5 mm. Moreover, eight, two, and ten patients did not meet the planning aim for the HRCTV_D90, whereas three, one, and three patients missed the planning limit with the systematic source position uncertainties of −5, 0, and +5 mm, respectively.

The variation of D_2cc_ with systematic source position uncertainty in ISBT for OARs is shown in Figure 5. The D_2cc_ for bladder (Figure 5a) and rectum (Figure 5b) decreased with an increase in positive source position shifts, whereas it increased for other OARs [bowel (Figure 5c) and sigmoid (Figure 5d)]. The D_2cc_ variation was statistically significant (*p* ≤ 0.003) at all source position uncertainties for all OARs. The mean D_2cc_ gradient was −78cGy/mm, 13cGy/mm, −57cGy/mm, and 72cGy/mm for bladder, bowel, rectum, and sigmoid, respectively. The D_2cc_ changed by 5.2 ± 4.6%, −1.3 ± 0.9%, 4.6 ± 2.5%, and −4.9 ± 2.4% at −5 mm and −5.0 ± 3.1%, 1.3 ± 0.9%, −3.9 ± 2.1%, and 6.6 ± 3.6% at +5 mm uncertainty for bladder, bowel, rectum, and sigmoid, respectively. The largest increases in D_2cc_ for bladder and rectum were 17.2% and 7.6%, respectively, at −5 mm, whereas they were 3.0% and 13.8% for bowel and sigmoid, respectively, at +5 mm. Moreover, the D_2cc_ exceeded the planning limit for bladder (one patient), rectum (one patient), and sigmoid (three patients) at −5, −1, and +5 mm source position uncertainty, respectively.

### 3.1 Re‐optimization of treatment plans

A comparison of dosimetric parameters from original and re‐optimized plans highlights the robustness of re‐optimized plans against source position uncertainty within the applicator. Table 2 presents the percentage difference in dosimetric parameters after targeted re‐optimization at source position uncertainty +5 or −5 mm.

(a) Re‐optimization of ICBT treatment plans

**TABLE 2:** Dosimetric impact of targeted re‐optimization.

Treatment type	Targeted OAR	Source position uncertainty (mm)	Bladder D_2cc_ % change^*^	Bowel D_2cc_ % change	Rectum D_2cc_ % change	Sigmoid D_2cc_ % change	HRCTV_D90 EQD_2_ % change
ICBT	**Bowel**	−5	−2.2	−**1.9**	−1.1	−0.8	−1.0
ICBT	**Rectum**	+5	−0.5	0.2	−**6.9**	0.04	−0.4
ICBT	**Sigmoid**	+5	0.5	0.6	−3.6	−**1.5**	0.7
ISBT	**Bladder**	−5	−**0.9**	0.9	0.4	0.6	1.6
ISBT	**Rectum**	−5	0.2	0.1	−**0.7**	0.1	0.6
ISBT	**Sigmoid**	+5	0.4	0.1	0.2	−**2.5**	0.4

*Note*: Bold letters and numbers indicate the targeted OAR and targeted dosimetric parameters, respectively.

* % change: Percentage difference in dosimetric parameters after re‐optimization (calculated using equation 3) of treatment plans at selected source position uncertainty.

**FIGURE 6:**
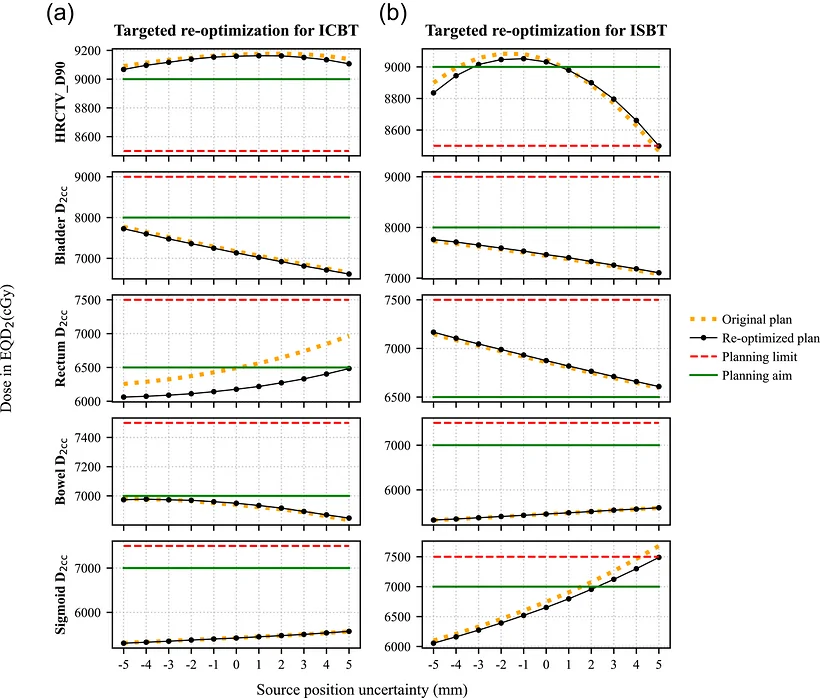
Two representative cases of targeted re‐optimization: Dosimetric parameters before and after re‐optimization to minimize (a) rectal dose for intracavitary brachytherapy (ICBT) and (b) sigmoid dose for interstitial brachytherapy (ISBT).

**FIGURE 7:**
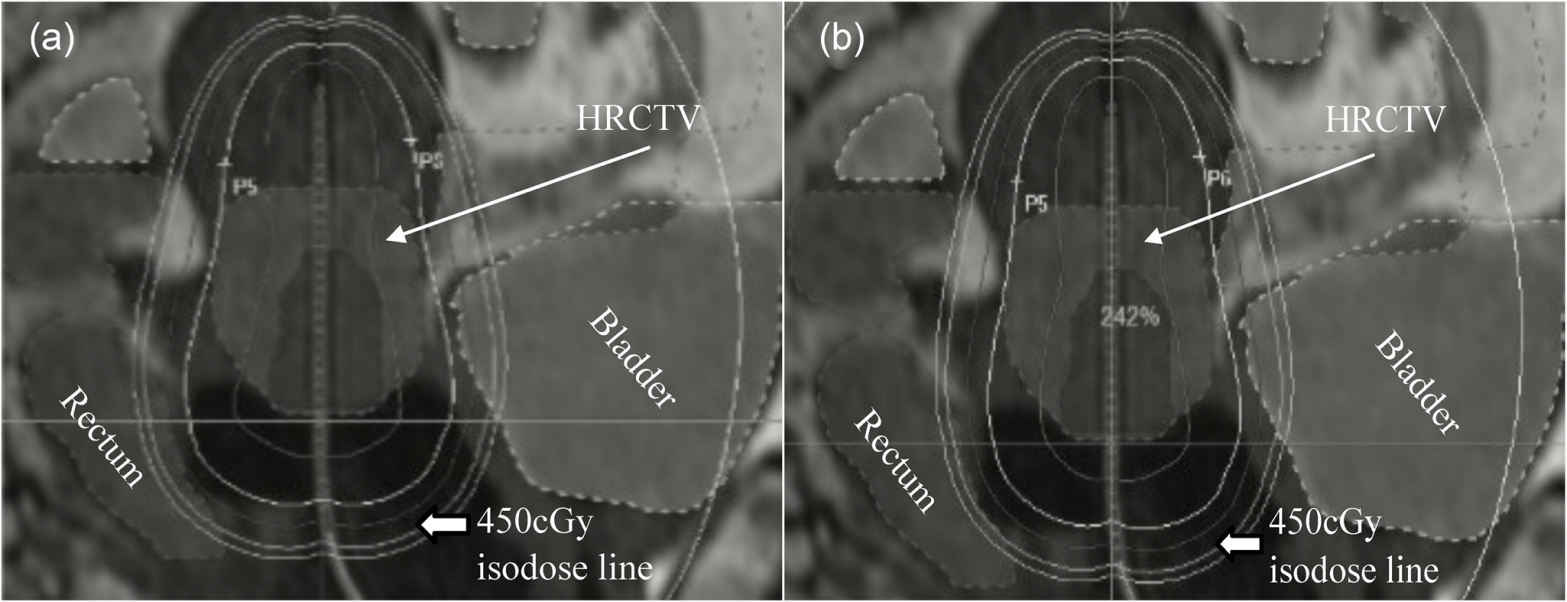
A sagittal MRI view displays isodose lines overlaid on the anatomical structures in intracavitary brachytherapy. (a) The initial treatment plan (b) The re‐optimized treatment plan.

The dosimetric impact of re‐optimization on ICBT treatment plans for three patients with the highest D_2cc_ for bowel, rectum, or sigmoid has been summarized in the first three rows of Table 2. The targeted re‐optimization successfully lowered the affected OAR D_2cc_ below the planning aim/limit while maintaining the HRCTV_D90, with a negligible change in the D_2cc_ of other OARs. Figures 6a and 7 show results (dosimetric parameters before and after plan re‐optimization) of the targeted re‐optimization for the rectum D_2cc_ reduction in ICBT. Moreover, for this representative patient, HRCTV_D90 EQD_2_ increased from 0 to +2 mm uncertainty in source position before a drop. The increase in dose was 0.07% according to the original plan and 0.02% according to re‐optimized plan at +2 mm shift in the source position. Figure 6a shows for one patient a 6.9% reduction in rectal D_2cc_ achieved with the HRCTV_D90 maintained. Moreover, with this re‐optimization, it was possible to lower rectum D_2cc_ below the planning aim. Figure 7a displays the initial treatment plan, where the 450cGy isodose line (shown with a thick white arrow) extends deeper into the rectal contour. The re‐optimization (Figure 7b) pushed isodose lines toward the outer boundary of the rectum, achieving substantial OAR sparing.

(b) Re‐optimization of ISBT treatment plans

**FIGURE 8:**
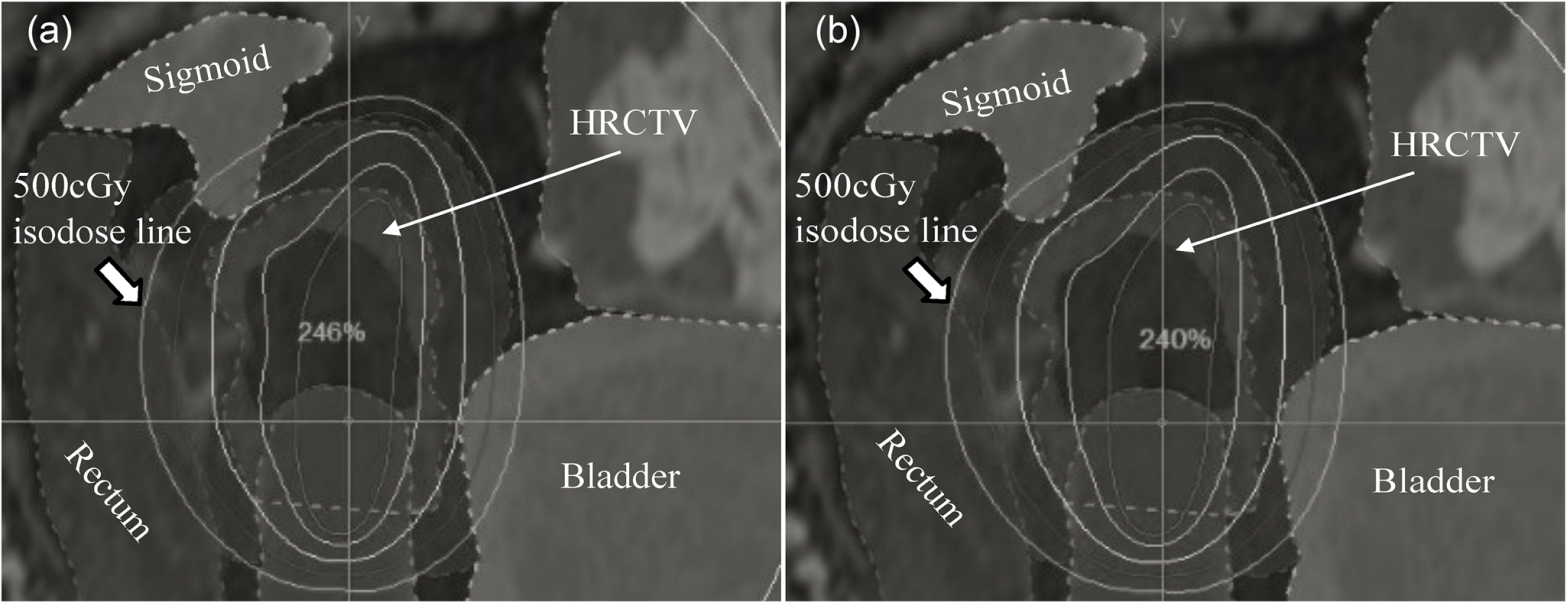
A sagittal MRI view displays isodose lines overlaid on the anatomical structures in interstitial brachytherapy. (a) The initial treatment plan (b) The re‐optimized treatment plan.

The dosimetric impact of re‐optimization on ISBT treatment plans for three patients with the highest D_2cc_ for bladder, rectum, or sigmoid has been summarized in the last three rows of Table 2. The targeted re‐optimization successfully lowered the affected OAR D_2cc_ below the planning aim/limit while maintaining the HRCTV_D90, with a negligible change in the D_2cc_ of other OARs. Figures 6b and 8 show results (dosimetric parameters before and after plan re‐optimization) of the targeted re‐optimization for the sigmoid D_2cc_ reduction in ISBT. Figure 6b shows for one patient a 2.5% reduction in the sigmoid D_2cc_ achieved with the HRCTV_D90 maintained. Moreover, with this re‐optimization, it was possible to lower sigmoid D_2cc_ below the planning limit. Figure 8a displays the initial treatment plan, where the 500cGy isodose line extends deeper into the sigmoid contour (shown with a thick white arrow). The re‐optimization (Figure 8b) pushed isodose lines toward the outer boundary of the sigmoid, achieving substantial OAR sparing.

## 4 DISCUSSION

Brachytherapy clinics employ various methods to minimize source position uncertainty through routine quality assurance practices.^32^ However, uncertainty in the source position within applicators because of applicator design,^17^, ^18^, ^21^, ^22^, ^23^, ^24^, ^25^ change in length of a source drive cable,^23^ and reconstruction in the applicator tip in MRI images^9^, ^26^, ^27^ can exist during treatment planning. The convergence of the above‐mentioned sources of uncertainty collectively compounds the overall systematic uncertainty of the source position within the applicator, and there is limited published data demonstrating the impact of such systematic uncertainty on treatment plans during MRI‐only cervical brachytherapy. In this paper, the dosimetric impact of the systematic source position shift within applicators during MRI‐only cervical HDR brachytherapy has been studied. To the best of our knowledge, this is the first study to evaluate the dosimetric effect of systematic source position shifts along the source path within the applicator during MRI‐only HDR cervical brachytherapy, using the Venezia tandem‐ovoid applicator for ICBT and interstitial needles with a tandem through transperineal template for ISBT. Moreover, this is the first study finding ways to minimize the impact of such uncertainty.

Modern 3D image‐based cervical brachytherapy plans use multiple applicators (like interstitial needles), and the dwell time modulation is larger compared to traditional standard plans that use very few channels. The impact of source position uncertainty within applicators can be expected to be significant for both OARs and the target in such modulated plans. Our study indicated that a shift of the order of millimeters of source position within the applicator is statistically significant for both ICBT and ISBT. Bladder D_2cc_ was most sensitive to the source position variation among all OARs, regardless of ICBT or ISBT. Rectum and sigmoid D_2cc_ gradients were larger in ISBT compared to ICBT. This variation was possibly due to a higher number of closer dwell positions in many channels in interstitial patients compared to the intracavitary applicators. An average increase in HRCTV_D90 EQD_2_ was observed for ICBT patients for +1 and +2 mm shift in source position. Our baseline planning utilizes standard library templates optimized to safely achieve standard prescription goals (8500cGy to 9500cGy EQD_2_ for HRCTV_D90) while prioritizing OARs sparing. Once these clinical goals are met, no further optimization is performed to maximize HRCTV_D90. When the source undergoes a positive shift, the high‐dose isodose lines are shifted superiorly toward the cranial portions of the HRCTV, mainly coming from tandem dwell shifts, resulting in an increase in HRCTV_D90. This effect is expected because the initial standard planning procedure is static and does not account for source positional uncertainty.

A previous study by Schindel et al.,^16^ for ICBT with MRI‐only 3D planning using a tandem and ovoid‐type applicator looked at two scenarios of the applicator reconstruction error: (a) tandem shifted in cranial direction with ovoids shifted in posterior direction and (b) tandem shifted in caudal direction with ovoids shifted in anterior direction. They found that the individual applicator reconstruction error of ± 4.5 mm resulted in a 2–3% change in HRCTV_D90, 4–6% in bladder D_2cc_, 3–4% in sigmoid D_2cc_, and 5–7% in rectum D_2cc_. They also looked at the whole applicator shift scenario in the cranial‐caudal direction and found that for the shift of ± 5 mm, the change in HRCTV_D90 was up to 5%, sigmoid D_2cc_ up to 5%, bladder D_2cc_ up to 8%, and rectum D_2cc_ up to 22%. The tandem shift in the above study resembles source position variation along the tandem axis in our study. However, the shift of the ovoid in a patient's cranio‐caudal direction or anterio‐posterior direction by Schindel et al.,^16^ differs from the source position variation in our study, which is along the curved source path of the ovoid applicators. Despite this difference, our dose difference results for ICBT plans were similar for HRCTV_D90 (up to 2%) and D_2cc_ for bladder (up to 6%), sigmoid (up to 2%), except for rectum (up to 2%). The large difference in rectum D_2cc_ is most likely because of the source position being very close to rectum when applicator shifts were simulated in the study of Schindel et al.^16^

Our results are also similar to the findings of Dookie, who reported ISBT to be more prone to under‐coverage of HRCTV for applicator displacement.^33^ Our study also indicates that ISBT is more prone to overdosing OARs in the presence of source position uncertainties. Dookie et al.,^33^ studied the effect of directional source position error by simulating systematic dwell position shifts up to ± 7 mm in the superior‐inferior, right‐left, and anterior‐posterior directions individually in HDR cervical brachytherapy. Although they studied both ICBT and ISBT cases, our study is close to their study for ISBT only with superior‐inferior source position shifts. Dookie et al.,^33^ in their study, found that a displacement of 4–6 mm was enough to trigger a 10% dose increase to bladder or rectum along with a compromise of 10% in HRCTV_D90. Our systematic source position shifts are not exactly in the same direction but closely resemble that of superior‐inferior shifts for ISBT cases. Our results of D_2cc_ for bladder (5.2 ± 4.6%) and rectum (4.6 ± 2.5%), and the drop in HRCTV_D90 (−6.2 ± 1.4%) with the systematic source position shift of 5 mm along the source path were similar. Moreover, inferior displacement is the most likely case of applicator displacement in a clinic. Our simulated source position uncertainty in the negative direction within applicators closely resembles with this clinical scenario for ISBT patients.

Our study showed that by selectively reducing dwell times, changing dwell time gradient, or even deactivating some of the dwell positions in source channels located near critical structures, the OAR D_2cc_ can be lowered, and their dosimetry can be made robust with no compromise in clinical target dose coverage. In ICBT, incorporating supplementary modulation and reducing dwell time in the proximity of the affected OAR or at the level of D_2cc_ reduced OAR D_2cc_. In ISBT, deactivating dwell position and removing or reducing modulation near the OAR was effective to reduce OAR D_2cc_. These differences in re‐optimization strategies between ICBT and ISBT should be due to the differences in the number and distribution of applicators between them.

Our findings indicate that uncertainty in the source position within applicators can result in a significant change in dosimetry. Moreover, the present study indicated that a selective re‐optimization has the potential to minimize the impact of source position uncertainty. The results of our study could be utilized in brachytherapy clinics to evaluate and minimize the impact of source position uncertainty during the treatment planning stage prior to the treatment delivery for similar applicators and patient cohorts. This strategy in the clinic will be particularly useful to the plans with the OAR dosimetry close to or exceeding the established planning aims or limits.

The uncertainty in source position within the applicator can exist during CT‐based planning as well. Thus, the findings of this study could be generalized to a CT‐based or hybrid CT/MRI treatment approach as well.

The main limitation of our study is the smaller number of patients. In the future, we plan to conduct a similar study with a larger cohort of patients to evaluate the impact of source position uncertainty and to test the statistical significance of dosimetric change, including during targeted re‐optimization. Moreover, re‐optimization of treatment plans may increase treatment planning time as it involves manual optimization, which could be crucial for some patients during intraoperative treatment. Furthermore, the targeted treatment plans were already highly modulated; therefore, re‐optimization strategies developed in this study may slightly increase dose to non‐targeted OAR or may not increase HRCTV_D90. During such a situation, a clinical decision needs to be made between robustness and dosimetric goals.

## 5 CONCLUSION

The present study shows that the systematic source position variation has a significant impact on OARs and target dosimetry in MRI‐only HDR cervical brachytherapy even in a small range of ± 5 mm. However, optimization of the treatment plan, that could be patient specific and mostly the applicator type dependent, could enhance its robustness against uncertainties in the source position for both target and OAR dosimetry.

## AUTHOR CONTRIBUTIONS

**Amritpal Singh**: Conceptualization; methodology; data collection and analysis; writing—original draft. **Raffi Karshafian**: Co‐supervision; writing—review and editing. **Ananth Ravi**: Co‐supervision; writing—review and editing. **Moti Raj Paudel**: Supervision; methodology; writing—review and editing.

## CONFLICT OF INTEREST STATEMENT

The authors declare no conflicts of interest.

## ETHICAL APPROVAL

The study has been approved by the research ethics boards of the Sunnybrook Health Science Centre and the Toronto Metropolitan University.

## Data Availability

The authors have nothing to report.
